# The relationship between *MUC5B* promoter, *TERT* polymorphisms and telomere lengths with radiographic extent and survival in a Chinese IPF cohort

**DOI:** 10.1038/s41598-019-51902-6

**Published:** 2019-10-25

**Authors:** Hui Wang, Yi Zhuang, Hui Peng, Min Cao, Yan Li, Qingqing Xu, Xiaoyan Xin, Kefeng Zhou, Geyu Liang, Hourong Cai, Jinghong Dai

**Affiliations:** 1 Department of Pulmonary and Critical Care Medicine, the Affiliated Drum Tower Hospital of Nanjing University Medical School, Nanjing, Jiangsu China; 2 Key Laboratory of Environmental Medicine Engineering, Ministry of Education, School of Public Health, Southeast University, Nanjing, Jiangsu China; 3 Department of Radiology, The Affiliated Drum Tower Hospital of Nanjing University Medical School, Nanjing, Jiangsu China

**Keywords:** Genetic predisposition to disease, Respiratory tract diseases

## Abstract

Genetic factors were identified to be associated with the development of idiopathic pulmonary fibrosis (IPF). We aimed to investigate associations between mucin 5B (*MUC5B)* and telomerase reverse transcriptase (*TERT*) polymorphisms and telomere length (TL) with honeycombing extent and survival in a Chinese IPF cohort. Seventy-nine patients diagnosed with IPF were enrolled. The honeycombing extents in high resolution CT scan (HRCT) were quantitatively scored and defined as mild (<10%), moderate (10–50%), and severe (>50%) upon the honeycombing extents involving the total lung. We tested five single-nucleotide polymorphisms [rs35705950, rs868903 in MUC5B, rs2736100, rs2853676 in *TERT* and rs1881984 in Telomerase RNA Gene (*TERC*) and TLs in peripheral blood leucocytes, and evaluated their associations with radiographic extent and survival in IPF. The minor allele frequencies (MAF) were significantly greater for *MUC5B* rs868903 (P = 0.042) and *TERT* rs2853676 (P = 0.041) in IPF than those in healthy controls. CT/CC genotype of *MUC5B* rs868903 (p = 0.045) and short TLs (p = 0.035) were correlated with the more extensive honeycombing opacities in HRCT. After adjustment for age, sex, and smoking status, *MUC5B* rs868903 polymorphism was the significant gene risk factors for reduced survival (p = 0.044) in IPF. *MUC5B* promoter rs868903 polymorphism and TLs were associated with radiographic extent and survival in a Chinese IPF cohort. These findings suggested a genetic clue for exploring the underlying molecular basis and pathogenesis of IPF.

## Introduction

Idiopathic pulmonary fibrosis (IPF) is a fatal interstitial pneumonia with unknown causes and a median survival of about 3 years^1^. Two new agents, pirfenidone and nintedanib, have been recommended for the treatment. However, these treatments could only slow disease progression and could not halt or reverse the fibrotic process^2^. According to the 2018 guideline, usual interstitial pneumonias (UIP) pattern with the presence of honeycombing in high resolution CT scan (HRCT) of the chest is critical for making a definite diagnosis of IPF^1^. The extent of honeycombing in HRCT is a predictive factor of mortality in IPF^3^.

The pathogenesis of IPF remains unclear and a detailed understanding of the molecular risk factors for IPF is urgently required. Genetic factors were identified to be associated with the development of IPF^4^. Prior studies indicated that the mucin 5B (*MUC5B*) promoter, telomerase reverse transcriptase (*TERT*) polymorphisms and Telomerase RNA Gene (TERC) contributed to disease susceptibility and prognosis in patients with IPF^5–7^, while most these studies were conducted in western countries, data in Asian populations were limited^8–11^. Furthermore, most studies focused on rs35705950 in *MUC5B* promoter polymorphism that was reported rare among Asian Ancestries^8^, while few studies investigated the associations in *MUC5*B rs868903 at chromosome 11p15. The association between *MUC5B* rs868903 and IPF was observed in an allelic testing and it was reported as a highly associated SNP in Genome-Wide Association Study (GWAS) discovery^10,12^.

IPF was also reported as the most frequent manifestation of telomerase-associated disease^13,14^. Telomerase gene mutations, which could result in telomere shortening, were considered as genetic risk factors in family pulmonary fibrosis. Shorter telomere lengths (TL) were attributed to the increased susceptibility and independently associated with reduced survival in IPF^7,13,15^. However, few studies evaluated the associations between gene risk factors and honeycombing extent in HRCT. Jonathan K. Alder *et al*. compared alveolar and lymphocyte telomere lengths from the same individuals and found that peripheral blood telomere may reflect alveolar epithelium telomere length^16^. Thus, we chose five SNPs, including *MUC5B* SNPs (rs35705950, rs868903), *TERT* SNPs (rs2736100, rs2853676) and *TERC* SNP (rs1881984) after searching the literature^5,12^, tested these SNPs and TLs in peripheral blood leucocytes, and investigated their associations with the radiographic extent of honeycombing in HRCT and survival in a Chinese cohort.

## Results

### Clinical data collection

In total, 93 patients diagnosed with IPF and 200 age- and sex-matched healthy controls from center of Health Examination of Nanjing Drum Hospital were identified from January 2014 to October 2017. Among IPF patients, 14 of them missing clinical data were excluded, 79 patients with complete clinical and radiographic information were enrolled. They were 68 (86.08%) males and 11 (13.92%) females with mean age of 64.19 ± 8.86 years (range from 37 to 81 years old). 42 (53.16%) patients had a history of smoking. All patients denied a family history or special occupational exposures. After reviewing HRCT appearances, 13 (16.46%) patients had a mild (<10%) extent of honeycombing proportion in HRCT, 34 patients (43.04%) had a moderate (10–50%) proportion and 32 (40.51%) patients had a severe (>50%) proportion. After admission, 21 (26.58%) patients received pirfenidone therapy, 15 (18.99%) patients received N-acetylcysteine (NAC) therapy alone, 26 (32.91%) received a combination of glucocorticoids and N-acetylcysteine therapy, one patient (1.27%) received nintedanib therapy. No treatment was performed in 16 patients (20.25%). Most of the patients who received glucocorticoids were initially given these therapies before definite diagnosis and glucocorticoids were stopped gradually after definite diagnosis. None patient in this cohort was performed lung transplantation because of the patients’ refusal.

The age- and sex-matched healthy controls included 200 self-reported healthy employees at the Center of Physical Examination of our hospital. This study was approved by Ethics Committee of Nanjing Drum Tower Hospital.

### Distributions of MUC5B and TERT allele frequency and gene polymorphisms in IPF and healthy controls

Hardy-Weinberg equilibrium (HWE) between healthy controls and patients was tested with the goodness-of-fit Chi-square Test. The C allele frequency of *MUC5B* rs868903 in IPF cohort was 55.06% which was significantly higher than those in control group (55.06% vs 45.40%, p = 0.042). The A allele frequency of *TERT* rs2853676 in IPF cohort was 12.05%, which was lower than that in control group (18.50%, p = 0.041). *MUC5B* and *TERT* genotypes were similar between IPF cohort and control group (Table 1). No significant differences of allele or genotype frequencies were observed in *MUC5B* rs35705950, *TERT* rs2736100 or *TERC* rs1881984.

**Table 1:** Characteristics of individuals in IPF group and control group.

		IPF group(n = 79)	Control group (n = 200)	P
Telomere length		1.8667 ± 0.7083	2.6979 ± 0.7520	<0.001^†^
***MUC5B*** **rs868903**				
Genotype	CC CT	22 (27.85%) 43 (54.43%)	38 (19.00%) 106 (53.00%)	0.107
	TT	14 (17.72%)	56 (28.00%)	—
	Total	79 (100.00%)	200 (100.00%)	—
Allele frequency	C	87 (55.06%)	182 (45.50%)	0.042
	T	71 (44.94%)	218 (54.50%)	—
	Total	158 (100.00%)	400 (100.00%)	—
***TERT*** **rs2853676**				
Genotype	AA AG	1 (1.27%) 16 (20.25%)	4 (2.00%) 66 (33.00%)	0.082
	GG	62 (78.48%)	130 (65.00%)	—
	Total	79 (100.00%)	200 (100.00%)	—
Allele frequency	A	18 (12.05%)	74 (18.50%)	0.041
	G	140 (87.95%)	326 (81.50%)	—
	Total	158 (100.00%)	400 (100.00%)	—

Data are mean (SD) or number (%). IPF = idiopathic pulmonary fibrosis. Genotype and minor allele frequency used χ^2^ test or Fisher’s exact test. ND means not done. ^†^Represented general Linear Model adjusted by age and sex.

### Association between TLs and gene polymorphisms with radiographic extent in HRCT

**Figure 1:**
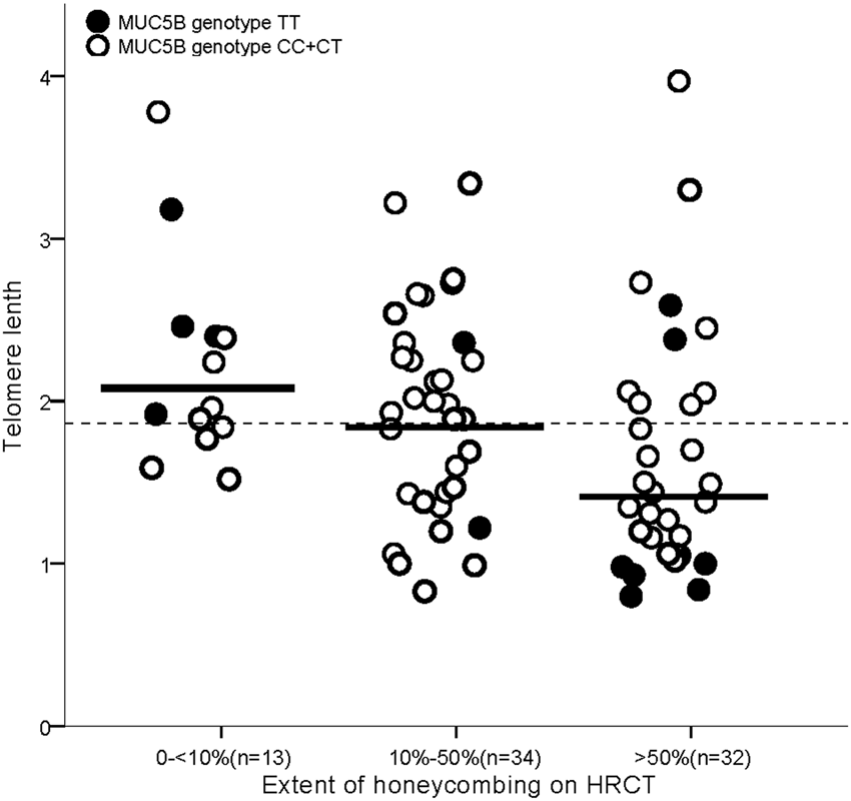
Distribution of polymorphism rs868903 and telomere length by HRCT. The distribution of *MUC5B* promotor single nucleotide polymorphism rs868903 polymorphisms and telomere length by radiographic extent of honeycombing. Data are presented for the IPF cohort. Horizontal lines show means of telomere length in each group. HRCT = high-resolution CT. Short TL was significantly correlated with the extent of radiological honeycombing in HRCT (p = 0.032). *MUC5B* rs868903 was associated with larger extent of honeycombing in patients with IPF (p = 0.045).

The mean TL in IPF patients was significantly shorter than that in healthy controls (mean TL: 1.8667 ± 0.7083 vs 2.6979 ± 0.7520, p < 0.001). When we stratified the patients according to the honeycombing extents in HRCT, the mean TL in mild extent of honeycombing group was 2.226 ± 0.644, the moderate group was 1.936 ± 0.638 and severe group was 1.647 ± 0.748. After adjustment of age and sex, short TLs were significantly correlated with the extent of radiological honeycombing in HRCT (p = 0.035, Table 2, Fig. 1).

**Table 2:** Mean telomere length in different range of honeycombing groups.

Extent of honeycombing	N	Mean of TL	95% CI	F	p	p adjusted
0~10%	13	2.226 ± 0.644	1.837~2.615	3.517	0.032	0.035
10%~50%	34	1.936 ± 0.638	1.714~2.159			
>50%	32	1.647 ± 0.748	1.377~1.916			
*Total*	79	1.867 ± 0.708	1.708~2.025			

Association between means of telomere length and extent of honeycombing in HRCT was evaluated by one-way ANOVA and General Linear Model adjusted by sex and age. TL = telomere length. <10%, 10–50%, >10% represent the range of honeycombing of the lung in HRCT.

A multivariate ordinal logistic regression analysis was performed to assess whether gene polymorphisms were independently associated with extent of honeycombing. After adjustment for age, sex and smoking status, CT/CC types of *MUC5B* rs868903 were significantly associated with larger extent of honeycombing in patients with IPF (p = 0.045, Fig. 1). No similar associations were found in *MUC5B* rs35705950, *TERT* rs2736100, rs2853676 or *TERC* rs1881984. The control group conformed to Hardy-Weinberg equilibrium (rs868903: p = 0.772 > 0.05; rs2853676: p = 0.566 > 0.05).

### Association of gene polymorphisms with survival

**Figure 2:**
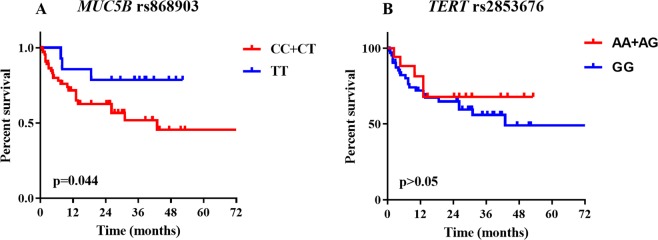
Survival in IPF patients. Overall survival was defined as the time from the date of diagnosis of IPF when they were identified to the date of death from any cause, with right censoring for individuals alive at the end of the study. Survival time was estimated for patients with IPF, stratified by genotypes. (**A**) Survival in *MUC5B* rs868903. Patients with CC + CT genotype of MUC5B rs868903 had significantly shorter OS than those with TT genotype of *MUC5B* rs868903 (p = 0.044). (**B**) Survival in *TERT* rs2853676.

Mean follow-up time was 30.36 months. Twenty-eight (35.44%) patients died during follow-up. Overall survival was defined as the time from the date of diagnosis of IPF when they were identified to the date of death from any cause. Mean overall survival (OS) was 23.66 months. Patients with CT and CC genotypes of *MUC5B* rs868903 had significantly shorter OS than those with TT after adjustment for age, sex, and smoking status (p = 0.044; Table 3, Fig. 2). Median OS was similar in patients among genotypes of *MUC5B* rs35705950, *TERT* rs2736100, rs2853676 or *TERC* rs1881984 (p > 0.05, Fig. 2).

**Table 3:** Cox proportional hazards analysis of survival in IPF group (n = 79).

	Univariate		Multivariate	
	HR (95% CI)	P	HR (95% CI)	P
Age (years)	1.018(0.970–1.068)	NS	—	NS
Sex (Female)	0.795 (0.240–2.639)	NS	—	NS
Even smoker	1.591(0.732–3.461)	NS		
Extent of honeycombing	2.464(1.302–4.662)	0.006	2.969(1.466–6.012)	0.003
**MUC5B rs868903**	—			
Variant vs Non-Variant				
TT	0.361(0.108–1.205)	0.098	0.280(0.081–0.965)	0.044
CT + CC				
**TERT rs2853676**	—			
Variant vs Non-Variant				
GG	0.683(0.260–1.799)	0.441	—	NS
AA + AG				

Multivariate COX regression included age, sex, extent of honeycombing and polymorphisms of *MUC5B* rs868903 or *TERT* rs2853676. NS = not significant.

## Discussion

To our knowledge, it is the first study to investigate associations between *MUC5B* rs868903 polymorphisms and TLs, and the radiological extents in a Chinese IPF cohort. The current study showed that both *MUC5B* promoter rs868903 polymorphisms and TLs in peripheral blood leucocytes were significantly associated with radiological features on the extent of honeycombing in HRCT. Moreover, our study firstly reported that MUC5B promoter rs868903 polymorphisms were associated with the reduced survival in a Chinese IPF cohort.

A series of studies had demonstrated that *MUC5B* might play a critical role in the pathogenesis of IPF^8–10^. In 2011, a large genome-wide linkage study evaluated the role of *MUC5B* promoter rs35705950 in the risk of IPF. Their results showed that the minor allele resulted in the increasing expression of *MUC5B* in the lung^10^. Meanwhile, genetic variants of *MUC5B* had also been identified in both Pittsburgh and Chicago cohorts^10^. These studies also suggested that dysregulated *MUC5B* expression in the lung might be involved in the pathogenesis of IPF. After that, a three-stage genome-wide association study (GWAS) have replicated the previously identified association between rs35705950 and IPF in European-American patients^17^. Carmel *et al*. also reported that *MUC5B* polymorphism rs35705950 was significantly associated with increased susceptibility to IPF in a UK population^11^. However, prior studies mainly focused on the associations between *MUC5B* rs35705950 and IPF. Few studies investigated another important SNP in *MUC5B* promoter (rs868903), which had been considered as one of the most strongly associated SNPs both in IPF and in IIP^12^. Moreover, prior studies just included patients from western countries. Data on these associations in East-Asia populations remained limited.

The current study, for the first time, demonstrated that *MUC5B* rs868903 polymorphism was associated with the extent of honeycombing opacities in HRCT and mortality in Chinese patients with IPF. Of note, no significant correlations between *MUC5B* promoter rs35705950 and IPF were observed, likely due to the small sample size and racial differences. Similarly, Peljto and co-authors found that *MUC5B* promoter rs35705950 polymorphism was very rare in Asian population^8^. According to the guideline^1^, honeycombing was a distinguishing feature of UIP pattern in HRCT which was the key appearance for the definite diagnosis of IPF. Pathologically, honeycombing in HRCT represented the abnormal tissue repair and progressive lung scaring formations following repetitive alveolar injury^18^. Our study provided a clue that *MUC5B* polymorphism may play a significant molecular factor in the disease development and outcome of IPF^19^. Further studies are needed to investigate the underlying mechanisms of *MUC5B* in the pathogenesis of IPF.

The field of telomere research had revealed that IPF was the most common manifestation of telomere-mediated diseases^14^. Telomerase gene polymorphisms have been reported to be associated with IPF in recent studies^5,14,17^. Previous work on the genetic basis of IPF showed that families with mutant telomerase genes displayed genetic anticipation and telomerase gene mutations were the commonest inherent risk factors of familial pulmonary fibrosis^7,20,21^. Our previous study also showed that telomerase gene mutations were associated with TL shortening and telomere shortening increased susceptibility and had valuable for predicting survival for patients with IPF^13^. In this study, we found that MAF of rs2853676 in *TERT* was significantly different between patients with IPF and healthy controls. Although no association was found between telomerase gene polymorphisms with IPF probably due to the small size sample, we observed that TL was associated with the extent of honeycombing in patients with IPF. These interesting findings argued that TL could participate the pathology of IPF. Ongoing investigations should be carried out to dissect the molecular mechanisms of telomere shortening in IPF.

The clinical course of IPF was highly heterogeneous and evaluation of the progression and mortality remained a great challenging. Traditional physiologic changes of lung function parameters in six months including forced vital capacity (FVC), forced expired volume in 1 second (FEV1), diffusion capacity for carbon monoxide of lung (DL_CO_) had been reported to provide accurate prognostic information^15,22,23^. In the genetic and molecular level, previous studies reported that TL was an independent risk factor of survival in patients with IPF^15^. *MUC5B* rs35705950 promoter was also reported to be strongly associated with the reduced survival of IPF^9,10^. In addition, several plasma proteins such as matrix metalloproteinase-7 (MMP-7), intercellular adhesion molecule-1 (ICAM-1), interleukin-8, and vascular cell adhesion molecule-1 (VCAM-1) had been evaluated as a tool for prognosis determination^24^. However, there was not a perfect predictive system generally acceptable so far. In this study, we firstly showed that *MUC5B* promoter rs868903 was associated with the radiographic fibrosis extent in HRCT and reduced survival in a Chinese IPF cohort. Taken together, our study provided a clue that an ideal predicting model should consider a combination of clinical, radiographic and genetic information, which enable better risk stratification and following personal treatment in future clinical trials.

There were several limitations in this study. First, the study cohort was from a single medical center with a small size sample for IPF. Second, based on the guideline^25^, typical UIP pattern in HRCT was sufficient for a definite diagnosis of IPF, this study only enrolled IPF patients with typical appearance of honeycombing in HRCT without pathology. There could be a selection bias toward patients with atypical CT finding of IPF. Third, a multi-disciplinary team diagnosis of IPF can be made without a lung biopsy^25^, and lack of lung biopsy restricted our ability to measure the expression of mucin and evaluate the association between *MUC5B* rs868903 polymorphism and the mucin expressions in lung tissue.

## Conclusion

Our study firstly reported that *MUC5B* promoter rs868903 polymorphism and TLs were associated with radiological features and *MUC5B* rs868903 polymorphisms was a predictive factor for the prognosis in a Chinese IPF cohort. Our study reinforced the conclusions in previous studies that *MUC5B* promoter and TL played an important role in IPF^9,10,13,15^. Further investigations are needed to determine exactly potential role of *MUC5B* gene in the pathogenesis of IPF and the regulation mechanisms of telomere shortening.

## Methods

### Patients cohort

The diagnosis of IPF was established in accordance with the criterion recommended by the 2018 international guideline^1^. Briefly, diagnostic criteria were the following items: (i) exclusion of other known causes of ILD (e.g., domestic and occupational environmental exposures, collagen vascular disease and drug toxicity) and either; (ii) the presence of UIP HRCT pattern not subjected to surgical lung biopsy; (iii) specific combinations of HRCT patterns and histopathology patterns in patients subjected to lung tissue sampling. Based on the criteria, patients who had typical UIP pattern on HRCT were included in the study. Patients who had known causes of pulmonary fibrosis were excluded.

### DNA extraction and polymorphisms examinations

Venous blood samples of all patients were collected at diagnosis with informed consent. The healthy controls were from center of Health Examination of Nanjing Drum Hospital. Genomic DNAs of IPF patients and healthy controls were extracted and purified from circulating leukocytes by using TIANamp Blood DNA Kit (Tiangen Biotech Co., Ltd., Beijing, China) and Genomic DNA Purification Kit (Life Technologies Corporation, Carlsbad, CA, USA). Sequencing reactions were induced by using a BigDye Terminator Cycle Sequencing Ready Reaction Kit (Applied Biosystems, Foster City, CA, USA). DNA samples were genotyped to identify SNPs associated with IPF by polymerase chain reaction (PCR). PCR conditions were available according to the requirements. Primers were designed by software PRIMER3 and were as the following: rs868903—forward, TGCAACACCAGCTCACCAT; reverse, GTCCACAGCAGCATCACT; rs1881984—forward, ATAATGAGAGTTCAGGGGA; reverse, ACAAAGCTAAAAGACAAGG; rs2736100—forward, AACATTGCTACCCTTGTCC; reverse, CTCCTCGTGAGTCTCCACA; rs2853676—forward, GTTCTCTGTGCCCTGAAGG; reverse, TGAAAGTGGCTGATGTTGA; rs35705950—forward, CCGCCCCTTTGTCTCCACT; reverse, ACTTTGCCCTCGTCCCTCC.

### TL measurement

Quantitative polymerase chain reaction (qPCR) was used to identify the relative repeat copy number of telomere and single gene (36B4a) with Power SYBR Green PCR Master Mix (Applied Biosystems, Carlsbad, CA, USA). Each reaction was performed in triplicate. The primer sequences were designed as the following: telomere—forward, ACACTAAGGTTTGGGTTTGGGTTTGGGTTTGGGTTAGTGT; reverse, TGTTAGGTATCCCTATCCCTATCCCTATCCCTATCCCTAACA; single gene (36B4a)—forward, CAGCAAGTGGGAAGGTGTAATCC; reverse, CCCATTCTATCATCAACGGGTACAA^1,15^. The standard curve was established by continuously diluting a reference DNA with deionized water into eight kinds of concentrations ranging from 1.875 to 240 ng/μL. Telomere/single copy numbers (T/S) ratio represented the relative telomere length of genomic DNA^13,26^.

### Radiographic evaluation

Chest HRCT scans of all patients were performed at end inspiration when they were enrolled. Based on prior study^27^, two dedicated chest radiologists (K.Z. and X.X.) reviewed the HRCT appearances respectively and quantitatively scored the extent of honeycombing involving as the percentage of total lung. The extent of honeycombing was defined as mild when it involved as <10% of total lung, moderate when involved 10–50% of total lung and severe when the honeycombing opacities involved >50% of the total lung. The variability of score by the two radiologists was evaluated and determined to be satisfactory for consistency^27^. Both radiologists were blinded to clinical and other demographic information.

### Survival analysis

All patients were regularly followed up every three to six months from the diagnosis. The evaluation in follow up included re-examination of chest HRCT and pulmonary function test. The primary endpoint was lung transplant or death of all causes. None of patients in this cohort received lung transplant. Survival time was defined as the interval from the date of diagnosis to death. Survival status was obtained from telephone interviews and the medical records. The follow-up time was censored at October 31, 2017.

### Statistical analysis

Minor allele frequency (MAF) and genotype frequency of SNPs were calculated and compared with Chi-square Test and Multiple Regression Analysis adjusted by age and sex. Associations between genotype polymorphisms and radiographic extent of honeycombing were assessed by multivariate logistic regression. The model was used to estimate odds ratios (ORs) and corresponding 95% confidence intervals (CIs) of rs868903 in *MUC5B* after adjusting for age, sex and smoking status. Association between TLs and extent of honeycombing in HRCT was evaluated by one-way ANOVA and General Linear Model adjusted by sex and age. All of the statistical analyses were performed by IBM SPSS Statistics (version 22.0J) and Graph Pad Prism 7.0. P value less than 0.05 was considered statistically significant for all tests.

## Ethical considerations

This study was approved by Ethics Committee of Nanjing Drum Tower Hospital. Written informed consents were obtained from all participants and all research was performed in accordance with the relevant guidelines/regulations.

## Data Availability

The datasets generated during and/or analysed during the current study are available from the corresponding author on reasonable request.
